# Electric Scooter Falls: The 2023–2024 Experience in the Clinical Emergency Children’s Hospital in Galați

**DOI:** 10.3390/clinpract14050145

**Published:** 2024-09-04

**Authors:** Cristina-Mihaela Popescu, Virginia Marina, Floriana Popescu, Andreea Oprea

**Affiliations:** 1 Dental-Medicine Department, Faculty of Medicine and Pharmacy, “Dunărea de Jos” University of Galati, 800201 Galati, Romania; cristina.popescu@ugal.ro; 2 Medical Department of Occupational Health, Faculty of Medicine and Pharmacy, “Dunărea de Jos” University of Galati, 47 Str. Domnească, 800201 Galati, Romania; 3 Department of English, Faculty of Letters, “Dunărea de Jos” University of Galati, 800201 Galati, Romania; floriana.popescu@ugal.ro; 4 Doctoral School, Faculty of Medicine and Pharmacy, “Dunărea de Jos” University of Galati, 800201 Galati, Romania; andreeapintilie4@gmail.com

**Keywords:** electric scooter, craniocerebral trauma, traumatic intracranial hemorrhage, pediatrics, emergency medical services

## Abstract

(1) Introduction: Since electric scooters were launched in 2017, they have become increasingly popular worldwide and a cause of childhood trauma. (2) Case reports: This paper has a double-fold purpose: it reports two cases of epidural hematomas and compares them with electric scooter-related head trauma in the literature. An overview of the literature on this topic was performed to make such a comparison. Our cases are one of almost 52 cm^3^ and one of 129 cm^3,^ both in two eight-year-olds. (3) Discussion: Although usually mild, traumatic brain injuries following e-scooter falls can also be moderate and severe. Reduced helmet use, high speed, and a lack of experience are the perfect set-up for potential severe injuries. Intracranial bleeds are not frequent, and epidural hematomas are rare in such cases, but they can significantly impact the individual, community, and healthcare system. No other medium- or large-sized epidural hematomas were reported in children sustaining electric scooter-related head trauma. (4) Conclusions: Our review parallels the literature and our hospital’s experience. Although there are both similarities and discrepancies between our cases and the literature, mild trauma should not be disregarded, for it may hide serious complications requiring immediate surgery.

## 1. Introduction

Pediatric head trauma is a widespread and severe issue in emergency rooms worldwide. In children, falls and motor vehicle accidents are the leading causes of head injuries [1,2,3,4].

As children grow and their mobility increases, i.e., in toddler and school-age children, the most frequent causes of head traumas include accidental trauma and traffic accidents [5]. Teenagers sustain sports-related head trauma and cycling or motorcycle riding [5]. These traumas can cause serious injuries that require surgical treatment, such as epidural hematomas (EDHs), depressed skull fractures, or penetrating skull injuries [5,6]. Different age groups use different two-wheeled vehicles, i.e., bicycles, scooters, and electric scooters (e-scooters).

According to the Safe Micromobility report published in 2024 [7], a standing electric scooter is a Type A micromobility vehicle equipped with a platform for the rider’s feet and an electric motor. It weighs under 35 kg and has a maximum powered design speed of 25 km/h [7].

Since electric scooters were launched in 2017, they have become increasingly used by adults, adolescents, and children [8,9], as they offer an inexpensive and easy way of transportation [9]. Conversely, shared e-scooter programs make them readily available through phone applications [10]. The demand for such vehicles has increased even more during the COVID-19 pandemic because they offer a way of transportation while simultaneously avoiding contact with other people [11].

This paper aims to answer the following questions: “How common are large epidural hematomas in pediatric patients following e-scooter falls?” and “Are our findings comparable with the literature?” To answer these questions, an extensive review of the PubMed, Scopus, and Cochrane databases was performed to identify the literature regarding pediatric epidural hematomas following electric scooter falls. Seven studies met the inclusion criteria, serving our comparative outlook. Thus, it both reports two large EDHs following e-scooter accidents observed in our hospital over the last three years, which were prepared following the CARE Guidelines [12] and narratively overviews the recent literature on EDHs in children related to standing e-scooter trauma.

## 2. 2023–2024 Epidural Hematomas Following E-Scooter Falls in the Clinical Emergency Children’s Hospital in Galați

### 2.1. First Case Report

An 8-year-old girl sustained accidental head trauma 24 h before coming to the emergency room of our hospital. She was brought to the hospital by her father in their car. Her main complaints were a head bump on the right side of the head and a headache. She told the emergency room doctors that she was riding an e-scooter without a helmet when she lost control of said vehicle and fell to the ground. She hit her head on the asphalt, did not lose consciousness, got on her feet, and decided to return home. For the past 24 h, she denied loss of consciousness, dizziness, or nausea but was feeling somnolent. The physical examination revealed a right parietal epicranial hematoma, a GCS of 14 points (E4V4M6). No other traumatic injuries were reported. She had no focal neurologic deficits or laboratory abnormalities, no known medical conditions, and was not on any medication. Her vital signs were normal.

A non-enhanced computer tomography (NECT) scan of the head was performed. It revealed a large temporoparietal hyperdense biconvex collection with small hypodense spots. It measured 6.7X1.5X cm (AXXAP). We calculated the cranio-caudal diameter using the Kothari formula [13] and obtained 10.7 cm. The bleed volume was also calculated using the Kothari formula ABC/2 [13] and had a volume of 52.43 cm^3^. The collection exerted a mass effect on the right parietal parenchyma, with decreased volume of the right lateral ventricle and a 5.8 mm midline shift to the left. Effacement of the right ambient cistern was present. Also, a linear overlying fracture with an epicranial hematoma was discovered. NECT images on this case are available in a pictorial review’s “epidural hematoma” section [14].

The final diagnosis was a right-sided temporoparietal epidural hematoma with active bleeding and mass effect. According to the guidelines, the neurosurgeon on call recommended immediate surgery [15]. Despite being informed of the risks and severity of the situation, the parents requested a transfer to another surgical facility. No therapeutic intervention or follow-up is available because the patient was transferred, but pharmacotherapy reduces morbidity and prevents complications [16]. The prognosis largely depends on the presentation time to the doctor and the patient’s comorbidities [17].

### 2.2. Second Case Report

**Figure 1:**
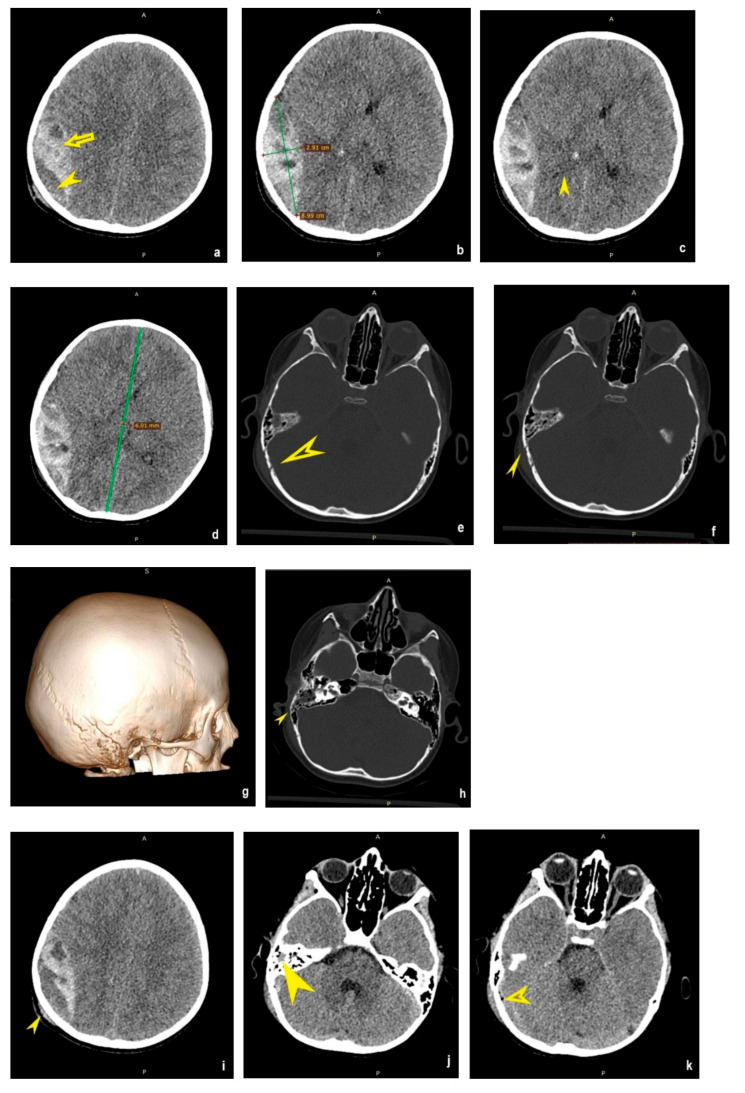
(**a**) Axial NECT brain window-inhomogeneous epidural hematoma with hyperdense areas (yellow arrow) and hypodense spots (yellow arrowhead) suggesting active bleeding; (**b**) axial NECT brain window with measurements; (**c**) axial NECT brain window-compressed right lateral ventricle-yellow arrowhead points to a fine hypodense line representing cerebrospinal fluid in the ventricle; (**d**) axial NECT brain window-midline shift to the left; (**e**) axial NECT bone window-yellow arrowhead pointing to the diastatic right arm of the lambdoid suture; (**f**) axial NECT bone window-solid yellow arrowhead pointing to the skull fracture; (**g**) VRT image-linear fracture parallel to the right arm of the lambdoid suture; (**h**) axial NECT bone window-yellow arrowhead pointing to the mastoid fracture; (**i**) axial NECT brain window-arrowhead pointing to the epicranial hematoma; (**j**) axial NECT brain window-arrowhead pointing to the hyperdense content of the right mastoid; (**k**) axial NECT brain window-arrowhead pointing to the small pneumocephalus. A—anterior; P—posterior; S—superior. The green lines it is antero-posterior diameter.

An 8-year-one-month-old boy was transferred from another hospital 70 km away to our Level III pediatric hospital. The ambulance staff informed our emergency team that almost two hours prior, he had sustained a fall from an e-scooter when trying to go up a hill that was covered in broken stone. The boy fell on his back and hit his right occipital area. He was not wearing a helmet. He was only accompanied by friends who ran to his house and alerted his mom, who called the ambulance. The boy lost consciousness but recovered shortly and did not remember what had happened. He was taken to the nearest hospital (adult-only hospital), the doctors on call checked him, and the transfer to our hospital was deemed necessary. He was brought to our emergency room. He was intravenously (IV) administered sodium chloride (0.9%) following the 4:2:1 rule. The clinical exam revealed that he had a right parietal-occipital hematoma, Glasgow Coma Scale score 10 (E3V3M4). He had no known medical conditions and was not on any medication. His vital signs (blood pressure, pulse, respiratory rate, and body temperature) were normal. On the AVPU scale, the child was V. Carbazochrome sodium sulfonate, ethamsylate, vitamin K, and tranexamic acid were administered IV. A NECT scan of the head was performed, and it revealed a large temporoparietal hyperdense biconvex collection with hypodense spots (Figure 1a). Maximal hematoma diameters in the axial plane were 2.9 × 9 cm (AXXAP) (Figure 1b). We calculated the cranio-caudal diameter using the Kothari formula [13] and obtained 9.9 cm. The bleed volume was also calculated using the Kothari formula ABC/2 [13], 129.19 cm^3^. The epidural collection had a mass effect on the underlying brain parenchyma, compressing the right lateral ventricle (Figure 1c) and shifting the midline 6 mm to the left (Figure 1d). Effacement of the basal cisterns was present. Near the diastatic right arm of the lambdoid fracture (Figure 1e)/linear temporoparietal fracture (Figure 1f,g) extending to the right mastoid process (Figure 1h), an epicranial hematoma overlies the fracture (Figure 1i). Hyperdense content of the right mastoid and middle ear (Figure 1j). Pneumocephallus near the mastoid (Figure 1k).

The final diagnosis was a large temporoparietal epidural hematoma with active bleeding and mass effect. Transfer to a neurosurgery hospital (300 km away) was decided, and the child was transferred by helicopter.

## 3. Discussion

Electric standing scooters were launched in 2017. In 2018, shared use of e-scooters began through sharing apps, which made them easily accessible, cheap, and a viable alternative to public transportation.

Standing electric scooters are becoming increasingly common not only in adults but alarmingly enough in children also [11,18,19,20]. Some reasons behind this include avoiding heavy traffic, eco-friendliness, and easy accessibility due to sharing apps. Using such micromobility vehicles increased during the COVID-19 pandemic and lowered in 2021 but increased afterward.

In Romania, electric scooters have been sold in stores since 2017, but the sharing system was implemented in the summer of 2020. At first, large cities had the sharing app available; in Galați (the fifth largest town in Romania), it became available in 2021. Increased e-scooter injuries have been reported since 2018, but mainly in adults [10,18].

Even though e-scooters have been accessible in Galați since 2018, intracranial bleeds are rare in the Clinical Emergency Children’s Hospital in Galați. There were only two such lesions in e-scooter victims in our hospital. Both of them were epidural hematomas, one medium-sized and one large. The severity of these injuries prompted a more in-depth search of the literature. To our surprise, we did not encounter pediatric studies focusing exclusively on head trauma from such accidents. At the same time, although children were included in cohorts alongside adults, no epidural hematomas of such size were mentioned.

Our review question was “How common are large epidural hematomas in pediatric patients following e-scooter falls?” We performed a search of the PubMed, Scopus, and Cochrane databases using “electric scooter” OR “e-scooter” OR “standing electric scooter” and “head trauma” OR “injuries” OR “e-scooter-related injuries” connected by the Boolean operator AND. PubMed retrieved 203 results, Scopus retrieved 304 results, and one result was found on Cochrane. One hundred ninety-three duplicates were removed, leaving 315 results for further investigation. Eligibility criteria included e-scooter accidents, epidural hematomas, and pediatric patients. We excluded papers that did not fit the criteria, leaving us 219 papers to review. We found seven eligible papers that focus exclusively on e-scooter-related head trauma in children. One of the reasons may be the current legislation that has been updated and limits children’s access to such vehicles. Another one could be that children are prone to musculoskeletal injuries in such cases, with a lower incidence of head trauma. Even so, when head trauma does occur in the pediatric age group, it is more commonly mild.

Morgan et al. reviewed pediatric e-scooter injuries in the UK in 2020 and identified ten patients, all with privately owned e-scooters [21]. This shows that e-scooter accidents have occurred since the launch of the micromobility. However, the alarming increase in e-scooter-related trauma is connected to sharing programs in the area [9,22,23]. Many e-scooter-sharing companies limit vehicle access to children under sixteen. Also, legislation in countries where e-scooters are available was updated, and children’s access was limited. Even so, there are still cases where underage children access such micromobility, and accidents occur. In Romania, the legislation on e-scooters was updated in 2024 [24]. According to the new law, children under fourteen are not allowed to ride such vehicles; helmet use is mandatory for people under sixteen riding on the road; and e-scooters should not be ridden on the sidewalk. There is only one person allowed to ride the e-scooter.

The demographic data of patients who are victims of e-scooter accidents varies throughout the literature. Morgan et al. reported that the patients were between 13 and 15 years old [21]. Cohen et al. analyzed a cohort of patients whose ages were over 3 [22]. Iriondo Muruzábal M et al. reported that the average age after the shared use of e-scooters is 5.6 and 13.7 years [9]. Saulitis included in their group four children with ages between 12 and 15 [20]. Hirsch reported e-scooter drivers to be over eight years old [25]. Bracher et al. included three patients aged 13, 15, and 16 [6]. Although legislation has changed over time, it is clear that some children ride e-scooters illegally. Unfortunately, both of our patients were eight years old. Their age is lower than that reported in the reviewed literature, and they are also under the age limit allowed by Romanian laws.

Most articles focusing on head trauma following e-scooter accidents mention a male predominance in pediatric cohorts [9,22,25]. However, male exclusivity was present in the study of Morgan et al. [21]. Female predominance was found in the pediatric cohort presented by Saulitis et al. [20], with three girls and one boy, while Bracher et al. [6] reported two girls and one boy. In our hospital, we found that epidural hematomas following e-scooter accidents were not exclusive to a gender in particular.

In pediatric e-scooter-related accidents, they happen because the driver loses control and falls, crashes with a vehicle, or hits a pedestrian [20]. The most common causes in this age group are losing control and falling. Cohen LL et al. hypothesized that the lack of experience and risk-taking attitude may cause such accidents [22]. The reduced ability to perceive hazards is also a cause at younger ages [21]. In both our cases, the mechanism of the trauma was falling from the scooter. This is similar to what was reported in the literature, falling being the most common cause of such trauma [21].

No matter how the accident occurs, risky and (later) illegal behavior has impacted traumatic e-scooter-related lesions throughout the years. In pediatric patients, risky behavior includes riding e-scooters and “forgetting” the helmet. Some studies reported that only one-third of the patients who were documented to have helmet use wore a helmet [22,25]. Morgan et al. found none of their patients wore helmets [21]. Cohen et al. reported pediatric helmet use at around 4%, which is still too low considering the impact potential traumatic brain injuries following e-scooter accidents can have on the life of the victim [22]. Neither patient wore a helmet in the cases we presented, which is consistent with part of the literature we reviewed [20,21].

Numerous studies have underlined the importance of helmet use and its reduced risk of head injury [6,8,20,26,27]. Wei W et al. and Fournier M et al. analyzed the impact of helmet-wearing on e-scooter-related head trauma [28,29]. Lab research using a hybrid III dummy [28] or a multibody model [29] underlines the importance of wearing a helmet [28]. The lab data revealed oblique head-ground impacts; the forehead was the first to hit the ground, Wei W et al. reporting it in 77.8% of their cases and Fournier M et al. in 44%. The oblique impact induces both linear and angular head acceleration. The latter is a critical factor in the severity of the traumatic injuries [28]. Helmets worn by patients and used in lab research were found to decrease the linear acceleration but not the angular one [28,29], thus protecting the head from mild TBI but not from severe TBI. Although helmet use was found to have limited protective roles, decreasing riding speed is a crucial component in preventing head injuries [28,29].

Illegal behavior, i.e., drivers under the legal age, alcohol consumption, and even recreational drugs, further increased the risk of developing traumatic injuries. Although it is unusual, there are reports of pediatric-age drivers arriving at the hospital intoxicated [8,9,21]. No alcohol or illegal substances had been consumed before riding the e-scooter by our patients.

In the northern hemisphere, most of the e-scooter accidents happen during the summer. Warm weather makes children go out and enjoy riding such micromobility vehicles [25]. Saulitis et al. reported pediatric cases between April and October [20]. The same tendency is noted in the southern hemisphere; only the months differ [30,31]. As presented in the literature, the e-scooter accidents in our cohort occurred during the year’s warm months in our region. The first epidural hematoma case was in September 2023, and the second one in May 2024.

The days when the most significant number of e-scooter-related trauma cases is Friday through Sunday [20]. Both accidents that led to the epidural hematomas we presented took place on Sundays; this coincides with the trend worldwide.

Although studies also mention the time of day when the accident occurred, nighttime is more common in teenagers and adults [32,33]. The cases we presented took place during the day, which is not unusual since they were both eight years old.

Studies reported limb and head trauma after an e-scooter accident. Children were found to experience musculoskeletal trauma [21] and polytrauma mainly [22]. Cohen et al. and Morgan et al. report more orthopedic injuries in pediatric patients, craniofacial injuries being the second most common lesions found in such patients [21,22]. Cohen et al. report in their cohort that 42.9% of the injuries are fractures, and 39.3% are head injuries. Both our patients only sustained head injuries, which is less common according to Cohen et al. [22].

Traumatic lesions following e-scooter accidents include contusions, concussions, skull fractures, and hematomas. Intracranial hemorrhages (subdural hematomas, traumatic subarachnoid hemorrhage, parenchymal hemorrhage, and epidural hemorrhage) were described only by Saulitis et al. [20]. Nevertheless, intracranial lesions may cause many disabilities and even deaths [34].

E-scooter-related head trauma is frequently mild [9]; this should not be taken lightly since even mild TBIs can develop long-term consequences [35], including post-traumatic stress disorder [21,36]. In our cases, one patient had sustained mild trauma (GCS 13–15), and one had a moderate TBI (GCS 10).

EDHs are extra-axial lesions commonly found in mild head trauma [37], but they can also appear in severe head trauma [38]. In children, such extra-axial bleeding can occur even in the absence of skull fractures [39,40].

One of our patients had GCS 10 and the other GCS 14. Both CT scans revealed large epidural hematomas that required surgical treatment.

Children are part of larger groups studied for head trauma following an e-scooter accident from a radiological [34,41,42] or neurosurgical point of view [23,43,44]; nonetheless, no pediatric study focusing exclusively on e-scooter-related head trauma was found. Even in studies that mention traumatic brain injuries after e-scooter accidents in pediatric patients, there is no mention of the volume of the hematoma. To our knowledge, only the study published by McKay W et al. mentions the volume of the hematoma found after e-scooter head trauma, and the average value was 17.8 cc (varying from traces to 125 cc) [45]. However, children and adults were included in the cohort, and there is no information on the ages of the patients sustaining such injuries. Saulitis et al. and Bracher et al. mentioned intracranial bleeding but not the volume [6,20].

## 4. Conclusions

A limited number of studies focus on e-scooter-related injuries exclusively in children. Cohen LL et al. focused on the epidemiology of admitted pediatric e-scooter injuries in patients under eighteen between 2015 and 2019 [22]. Morgan C et al. published a case series and review of the literature on pediatric e-scooter injuries in the UK in 2020 [21]; McGalliard R et al. published an analysis on pediatric e-scooter injuries over two years [46]. Iriondo Muruzábal M et al. present the demographical characteristics and number of pediatric patients consulted in the emergency room after an e-scooter-related TBI before and after large-scale use of e-scooters [9]. Hirsch et al. focused on cranio-facial fractures in children riding modern recreational conveyances, including e-scooters [25]. Bracher et al. described trauma characteristics following e-scooter trauma [6].

Our experience with head trauma following e-scooter trauma is limited. We only encountered two epidural hematomas in all the years following e-scooter sharing apps. There are similarities and discrepancies between our patients and other cases or studies we discovered in the literature. To the best of our knowledge, no epidural hematomas mentioned in the literature are as large as the ones we showcased.

Although e-scooters are easily available to children and may represent a means of having a good time or enjoying nice weather with friends, riding such a micromobility should not be taken lightly. The risk of sustaining head, orthopedic, or polytrauma injuries should be taken into account when thinking about taking a ride. Abiding the law—not riding under 16 and wearing a helmet—should be the norm in such cases. Regarding younger children, parents should be informed of the catastrophic consequences such as riding pose. Authorities should organize campaigns and implement strategies to raise awareness among children and adults of the risks of riding e-scooters. Also, the voice and experience of health providers should weigh more when designing and implementing campaigns. Authorities should remember that patients are not just numbers in statistics but individuals whose lives do matter.

## Data Availability

The dataset is available on request from the authors.
